# A novel CD4 knockout mouse strain with a spontaneous frameshift mutation in the CD4 locus

**DOI:** 10.1371/journal.pone.0266589

**Published:** 2022-04-06

**Authors:** Mathangi Janakiraman, Shin-Young Na, Gurumoorthy Krishnamoorthy

**Affiliations:** Research group Neuroinflammation and Mucosal Immunology, Max Planck Institute of Biochemistry, Martinsried, Germany; Hirosaki University Graduate School of Medicine, JAPAN

## Abstract

T cells express co-receptors CD4 and CD8, which are involved in the recognition of antigen presented to T cell receptors. The expression of CD4 in thymic hematopoietic cells is crucial for the thymic development and selection of T cells. In this study, we identified a novel CD4 mutant allele that emerged spontaneously in our mouse colony. The frameshift mutation led to a truncated CD4 protein which failed to reach the plasma membrane resulting in impaired development of CD4^+^ helper T cells. The CRISPR mediated correction of mutant allele restored the membrane CD4 expression. Further, using an adoptive transfer of T cells, we show that this model is an ideal recipient mouse for the study of CD4^+^ T cells.

## Introduction

The commonly used inbred mouse strains in the research were generated from a small founder population that was selectively maintained via brother-sister mating for extended periods [1]. As a consequence, the genetic diversity in these mouse strains is extremely limited compared to wild mouse populations. Nevertheless, genetic variations among the mouse strains lead to distinct haplotypes related to specific variations in certain genomic regions. For example, a mutation in *Tlr4* in C3H/HeJ mice renders this strain resistant to endotoxin [2]. The widely used congenic CD45.1 mouse is also shown to have mutations in genes *Sox13* and *Ncr1* which have consequences on their susceptibility to infections [3, 4]. Although the majority of the genetic variations can be attributed to the small founder population, further genetic drift also occurs in inbred mouse strains [5]. Spontaneous mutations are indeed detectable as random genetic events in many colonies of mice and there are numerous spontaneous mutation-derived models which are suitable for human disease research [6].

CD4^+^ helper T cells play a crucial role in adaptive immune responses. It is well established that the expression of the CD4 gene plays an important role in T cell development within the thymus and subsequent recognition of antigen presented in the context of MHC class II [7]. The committed lymphoid progenitors that arise from the bone marrow lack the expression of CD4 and CD8 (double negative; DN) and they reach the thymus for further differentiation. During the early developmental stage, these committed lymphoid progenitor cells undergo extensive cell division and a series of recombination events to rearrange their T cell receptor. At this time, they also start to express CD4 and CD8 co-receptors to form a population of CD4^+^CD8^+^ double-positive (DP) αβ-TCR-expressing immature cells that constitute more than 90% of the thymic cell population. The subsequent positive and negative selection events require cognate interactions of TCR with self-peptide-MHC ligands thereby selecting the T cells that are best suited for the host. The CD4 co-receptor expression plays an important role by ligating with MHC class II to provide survival signals that are essential for T cell development [7]. Hence, it is not surprising that the lack of expression of co-receptors curtails the T cell development in the thymus [8, 9].

In this study, we describe a new CD4 mutant mouse strain (CD4^fs^) from our mouse colony that lacks expression of the CD4 gene due to a spontaneously arising frameshift mutation in the CD4 gene. We characterize this new model and in further experiments, we show the suitability of this new mutant mouse strain as a recipient for CD4^+^ T cell adoptive transfer experiments.

## Material and methods

### Mice

β2m –/–mice (B6.129P2-*B2m*^*tm1Unc*^/J) [10] were originally obtained from Jackson Laboratory and maintained in the animal facilities of the Max Planck Institute of Biochemistry, Martinsried. Routine genotyping of β2m –/–mice was performed from tail DNA using primers (oIMR184—TAT CAG TCT CAG TGG GGG TG; oIMR185—CTG AGC TCT GTT TTC GTC TG; Neo sense—GGA TTG CAC GCA GGT TCT CCG). To generate F1 mice, β2m –/–mice were bred with wild-type (WT) C57BL/6. F2 mice were produced by brother-sister mating of F1 mice. CD4 mutant mice (CD4^fs^) were genotyped by Sanger sequencing of the PCR products generated from tail genomic DNA (CD4-forward–CTGCCAGAGTAGCGATCGAG; CD4-reverse–GCCCTCTCGTAAACTGTGCT). Animal protocols were approved by the Regierung von Oberbayern (Munich, Germany).

### Cell isolation and flow cytometry

Single-cell suspensions were prepared from the spleen or thymus by mechanical disruption via forcing through 40 μm cell strainers. Cells were collected in RPMI (RPMI 1640, Sigma Aldrich) containing 10% heat-inactivated Fetal Bovine Serum (FBS, Sigma Aldrich). Erythrocytes were depleted by resuspension in erythrocyte lysis buffer (0.83% NH_4_Cl) and incubation for 3 min at room temperature. The lysis buffer was then neutralized with RPMI containing 10% FBS and the cells were washed and collected in FACS buffer (PBS + 1% BSA) for staining. For peripheral blood mononuclear cell isolation, one drop of blood was collected by retro-orbital bleeding into heparin-containing tubes. The blood was incubated with erythrocyte lysis buffer (150 mM NH_4_Cl, 1 mM KHCO_3_, and 0.1 mM EDTA) for 5 min at room temperature, after which the cells were pelleted and resuspended in FACS buffer for staining. The following antibodies were used for staining: TCR-β-FITC, H2-Kb-APC, CD19-PE, CD4-Percp-cy5.5, I-A/I-E-BV421, CD8-BV605. All antibodies were purchased from Biolegend. Stained samples were acquired on the FACS Canto (BD biosciences). Data were analyzed using FlowJo (Treestar).

### Cell transfer and proliferation assay

Single-cell suspension of splenocytes isolated from myelin-specific TCR transgenic mice [11] was washed once with PBS and then resuspended in a solution of 1:250 diluted 5 μM eFluor450 dye (Biolegend) in PBS. Cells were then incubated for 10 min at room temperature after which RPMI containing 10% FBS was added. Cells were then washed and resuspended in PBS and injected intravenously at a concentration of 3–4 million cells per mouse, into CD4^fs^ mice. After 24 h, the CD4^fs^ mice were immunized by subcutaneous injection of 100 μg of MOG 35–55 peptide with 5 mg/ml of CFA. After 72 h, inguinal lymph nodes were collected from the mice and single-cell suspensions were prepared. Cells were stained with CD4-PE, CD45.2-APC, Vβ11-Percp-cy5.5, Vα3.2-FITC, and Fixable viability dye eFluor780, and acquired on the FACS Canto (BD biosciences). Data were analyzed using FlowJo (Treestar). Proliferation was normalized to that of WT mice.

### PCR and sequencing

CD4 gene was sequenced as described in genotyping. RAG genes were amplified by PCR and sequenced with the following primers: Rag1-forward–TGTTCCCAGGTAGCTTAGCCAACATG, Rag1-reverse–CCTCACTGCAACCCAAAGGAAAACAC; Rag2-forward–AAAGACCTATTCACAATCAAAAATGTCC, Rag2-reverse–TCAGAGAGCAATATACCTGAGTCTGAG.

### Plasmid construction and retroviral infections

To express wild type and mutant CD4, the entire coding sequence was amplified from the thymus cDNA from wild type and CD4 mutant mice with the following primers: CD4-forward–TCAGATTCCCAACCAACAAGAGCTC and CD4-reverse–GCCCTCTCGTAAACTGTGCT. The purified PCR products were cloned into a retroviral vector pMSCV-IRES2-BFP digested with the Hinc II enzyme. Colony PCR was performed for obtaining the correctly cloned constructs. The final construct was further verified by Sanger sequencing.

### Retrovirus supernatant production and transduction

Replication-deficient Phoenix ecotropic retrovirus packaging cells were transfected with 12 μg of pMSCV retroviral vector and 3.5 μg of pCL-Eco packaging plasmid by calcium-phosphate/chloroquine method. The virus-containing culture supernatant was collected 48 h and 72 h later. The virus supernatants were filtered through a 0.45 μm filter and supplemented with polybrene at 8 μg/ml. 2 ml of virus supernatant was added to 500,000 58 –/–cells seeded in a 6 well plate and centrifuged at 2000 rpm at room temperature. The medium was replaced after 24 h and the transduced cells were analyzed by flow cytometry after 48 h. BFP^+^ cells were sorted using FACS Aria II to establish a stable cell line.

### CRISPR knock-in

The CRISPR RNA (crRNA) targeting the CD4 gene (CD4 crRNA -AGGATCCAGGTTTTATCCAG) and the homology-directed repair (HDR) ssDNA oligo template (GTGATAAGGTCAAGATGGACTCCAGGATCCAGGTTTTATCCAGAGGGGT GAACCAGACAGTGTTCCTGGCTTGCGTGCTGGGTGGCTC) was designed using the online platform of IDT technologies. crRNA was ordered from Integrated DNA Technologies in their proprietary Alt-R format. To prepare the duplex, CD4 crRNA and Alt-R tracrRNA (1072534; IDT Technologies) were reconstituted to 100 μM with Nuclease-Free Duplex Buffer (IDT). Oligos were mixed at equimolar concentrations in a sterile tube (5 μl Alt-R crRNA and 5 μl Alt-R tracrRNA). Oligos were annealed by heating at 95°C for 5 min and slowly cooling down to room temperature. The crRNA–tracrRNA duplex (0.5 μl) and 1 μl NLS-Cas9 Protein (core facility MPI Biochemistry) were gently mixed by pipetting and incubated at room temperature for 20 min.

58 –/–cells were washed once with PBS and 5 x 10^5^ cells were resuspended in 10 μl buffer R (Thermo Fisher Scientific). 1.5 μl crRNA–tracrRNA duplex, 0.5 μl electroporation enhancer (1075915; IDT technologies) and 0.5 μl of 100 μM HDR template was added to the cells and gently mixed. The mixture was taken into 10 μl Neon electroporation tip and electroporated (1600V, 3 pulses, 10ms) using Neon transfection device (Thermo Fisher Scientific). Transfected cells were immediately transferred to a pre-warmed medium containing Alt-R-HDR enhancer (IDT Technologies).

### Exome sequencing

Whole exome sequencing and bioinformatics analysis were performed by BGI Tech Solutions (Hong Kong). Briefly, tail genomic DNA was randomly fragmented by sonication and the fragments were size selected for containing 200 bp– 300 bp. Then adapters were ligated to both ends of the resulting fragments. Extracted DNA was amplified by ligation-mediated PCR, purified, and hybridized to the exome array for enrichment. Captured products were subjected to Agilent 2100 Bioanalyzer and quantitative PCR. Each qualified captured library was then sequenced on Illumina Hiseq. Sequencing-derived raw image files were processed by Illumina base-calling software for base-calling with default parameters and paired-end reads were generated.

The clean data was produced by filtering raw data and the clean reads were mapped to the mouse reference genome using Burrows-Wheeler Aligner (BWA software [12]. To ensure accurate variant calling, the recommended Best Practices for variant analysis with the Genome Analysis Toolkit (GATK, https://www.broadinstitute.org/gatk/guide/bestpractices) was followed. Local realignment around InDels and base quality score recalibration was performed using GATK [13, 14], with duplicate reads removed by Picard tools (http://broadinstitute.github.io/picard/). All genomic variations, including SNPs and InDels, were analyzed using HaplotypeCaller of GATK(v3.6). After that, the hard-filtering method was applied to get high-confident variant calls. Then the SnpEff tool (http://snpeff.sourceforge.net/SnpEff_manual.html) was applied to perform a series of annotations for variants.

### Statistical analysis

Statistical analysis was performed using GraphPad Prism v9. Data are represented as mean ± s.e.m. Mann—Whitney’s U test was performed on the data and p-value > 0.05 was considered significant.

## Results and discussion

### A spontaneous mutant mouse with CD4 T lymphocyte deficiency

**Fig 1:**
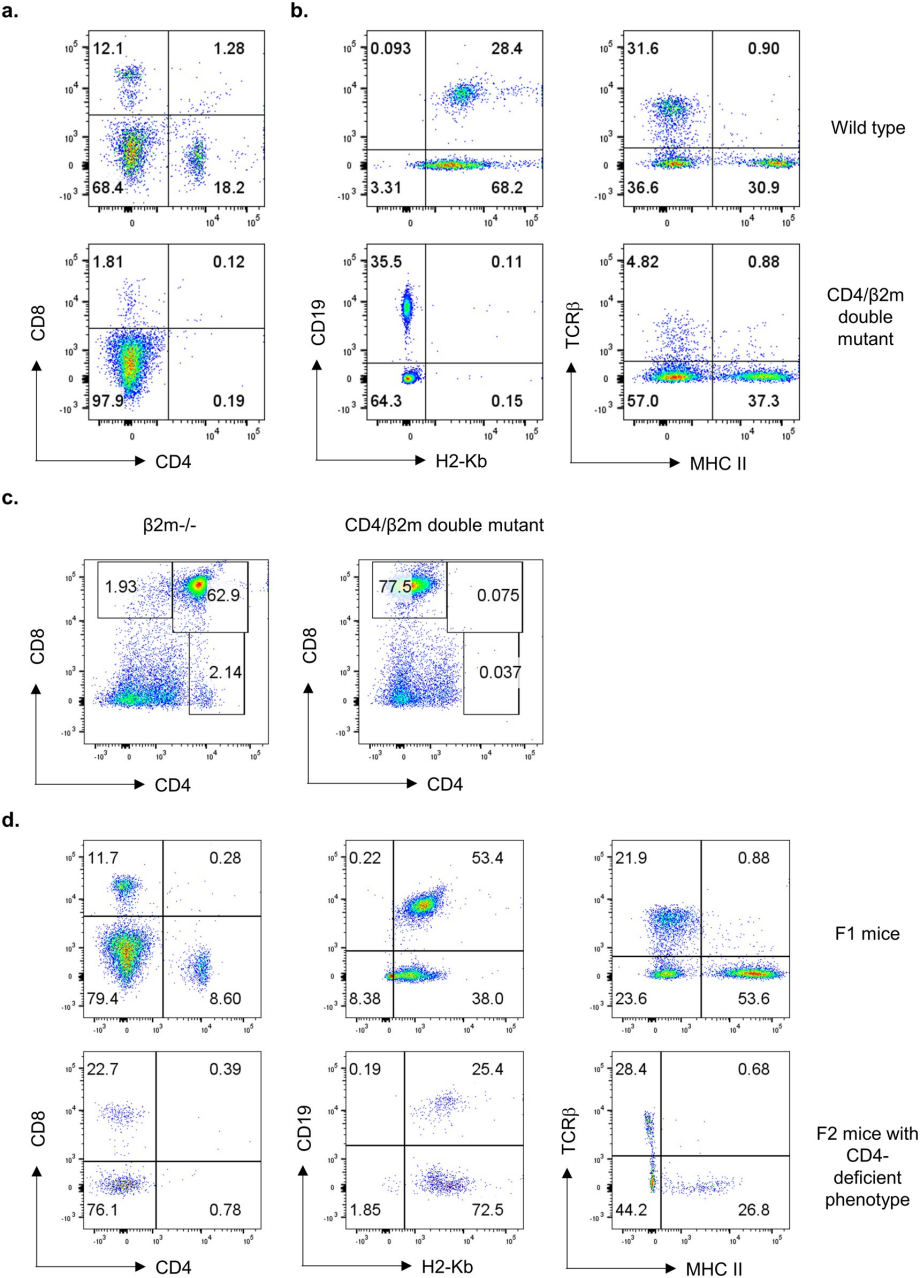
Characterization of spontaneous CD4-deficient phenotype. **(a–b)** Flow cytometry analysis of peripheral blood mononuclear cells from wild type (top) and β2m –/–mice with CD4-deficient phenotype (bottom). Peripheral blood cells were stained with anti-CD4, anti-CD8, H2-Kb, CD19, TCRβ, and I-A/I-E antibodies. Representative flow cytometry plots from 6 independent litters of mice that were analyzed are shown. n = 6–21 mice per group. **(c)** Flow cytometry analysis of thymocytes from β2m –/–mice (left) and β2m –/–mice with CD4-deficient phenotype (right). Thymocytes were stained with anti-CD4 and anti-CD8 antibodies. Representative flow cytometry plots from at least 4 independent experiments are shown. n = 6–44 mice per group. **(d)** Flow cytometry analysis of peripheral blood mononuclear cells from F1 mice (top) and F2 mice (bottom) from the breeding of CD4-deficient phenotype with wild type C57BL/6 mice. Peripheral blood cells were stained with anti-CD4, anti-CD8, H2-Kb, CD19, TCRβ, and I-A/I-E antibodies. Representative flow cytometry plots 2 independent experiments are shown. n = 4–8 mice per group.

While inbred mouse strains such as C57BL/6 mice are almost genetically identical, there are spontaneous mutations that occur in the genome resulting in breeding colony-specific sub-strains with potentially unique mutations across the genome. During the routine phenotyping of our mouse colony, we observed that a proportion of mice that are deficient for the β2m gene lacked not only CD8^+^ T cells but also CD4^+^ T cells in the blood (Fig 1a). This is unexpected since β2m –/–mice lack MHC class I (H2-Kb) but have intact MHC class II which is required for the proper development of CD4^+^ T cells. We confirmed that these mice had normal MHC class II expression in antigen-presenting cells suggesting that the lack of CD4^+^ T cell development is not due to abnormal expression of MHC class II (Fig 1b). Apart from MHC class II, mutations in the recombination activating gene (RAG) could also affect the development of CD4^+^ T cells. To examine whether unexpected random mutations within the RAG genes could be the reason for this phenotype, we sequenced the RAG genes from genomic DNA as well as RNA isolated from the thymus. The sequencing results showed that there are no unexpected mutations in the *Rag1* or *Rag2* genes. Also, the presence of intact CD19^+^ B cells in these mice suggested that the functional RAG genes are expressed in this mouse strain, and the CD4 T cell deficiency is not due to alterations in the RAG gene expression (Fig 1b). However, there is a small population of circulating TCRβ^+^ population (~5%) in CD4/β2m double mutant mice suggests that some class II-restricted T cells might have been positively selected and populated peripheral immune niches (Fig 1b). This is consistent with previous studies which showed the existence of MHC class II-restricted T cell populations in the periphery of CD4 knockout mice [15, 16]. Further, flow cytometric analysis of the thymus of these mice showed an abnormal thymic profile compared to β2m –/–mice. While the β2m –/–mice have normal CD4^+^CD8^+^ double-positive (DP) and CD4 single-positive population, the double mutant mice completely lacked DP and CD4 single-positive populations and there was an increase in the CD8^+^ single positive cell population (Fig 1c).

### CD4 T lymphocyte deficiency is a recessive phenotype

To identify whether the CD4^+^ T lymphocyte deficiency is a dominant or recessive phenotype, we crossed the β2m –/–mice with wild-type C57BL/6 mice to generate F1 mice. Analysis of the peripheral blood cells showed that all F1 offspring mice had a normal frequency of CD8 T cells and B cells, and a reduced frequency of CD4 T cells suggesting that the observed phenotype is recessive (Fig 1d). The reduced CD4 T cell frequencies could be due to lower expression of CD4 on their surface in heterozygous mutant mice compared to wild type (mean fluorescence intensity of CD4 staining: CD4 +/+: 14940 ± 704 vs CD4 +/-: 11190 ± 430). As expected, the expression of MHC class I and MHC class II in the F1 mice were also normal (Fig 1d). We then intercrossed F1 mice with each other and generated 42 F2 offspring mice. Flow cytometry analysis of these mice showed that 5 out of the 42 mice had the CD4 deficient phenotype but had normal CD8^+^ T cells and CD19^+^ B cells with wild type β2m gene (Fig 1d). These results suggested that the CD4-deficient phenotype is recessive and not associated with the β2m –/–phenotype.

### Exome sequencing identifies an indel mutation in the CD4 transmembrane domain

**Fig 2:**
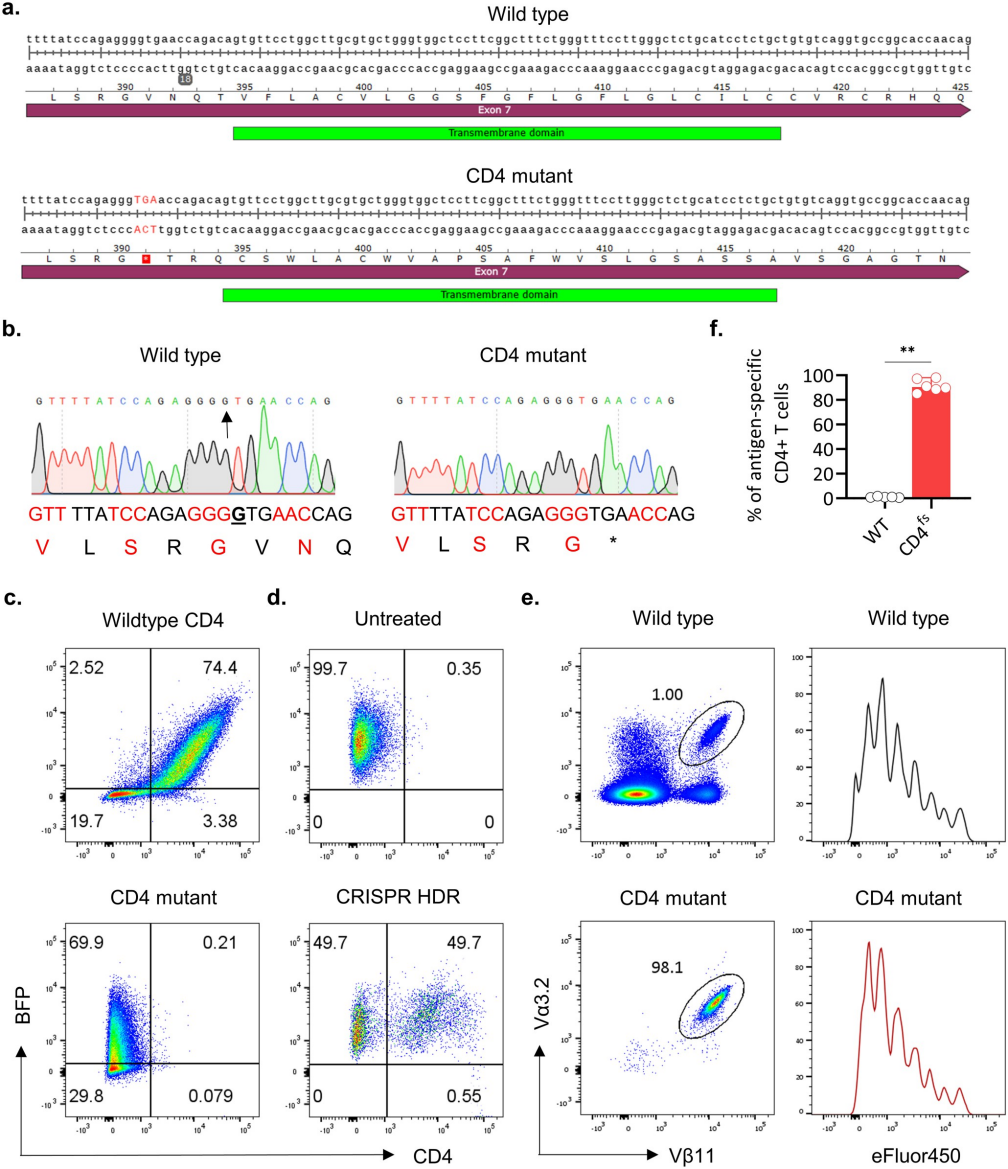
Identification of the mutation responsible for the CD4-deficient phenotype. **(a)** Indication of the indel mutation leading to a frameshift in the CD4 mutant mice, resulting in a premature stop codon in the exon 7, identified through exome sequencing. Sequences of the exon 7 from both wild-type mice (top) and CD4 mutant mice (bottom) are shown, with the frameshift resulting in a stop codon highlighted in red. **(b)** Sanger sequencing of the CD4 gene from tail genomic DNA in wild-type mice (left) and CD4 mutant mice (right), confirming the presence of the mutation at this site. Representative sequencing data from 6 independent litters of mice that were analyzed are shown. n = 10–24 mice per group. **(c)** Flow cytometry analysis of 58 –/–cells transduced with wild type (top) or mutant CD4 gene (bottom) with a retroviral vector expressing BFP. 58 –/–cells were stained with anti-CD4 antibody and the expression of both BFP and CD4 was observed. Representative flow cytometry plots from 2 independent experiments are shown. **(d)** Flow cytometry analysis of BFP^+^ 58 –/–cells transduced with the CD4 mutant construct and untreated (top) or receiving the Cas9/sgRNA/repair nucleotide complex (bottom). 58 –/–cells were stained with anti-CD4 antibody and the expression of both BFP and CD4 was observed. Representative flow cytometry plots from 2 independent experiments are shown. **(e–f)** Flow cytometry analysis of inguinal lymph nodes from wild type (top) or CD4^fs^ (bottom) mice after adoptive transfer of 3–4 million eFluor450 labeled cells from myelin-specific TCR transgenic mice. Mice were immunized with 100 μg MOG 35–55 in 5 mg/ml of CFA 24 h after cell transfer and lymph nodes collected 72 h after immunization. **(e)** Representative flow cytometry plots show the transferred Vα3.2^+^ Vβ11^+^ T cell population (left) and the dilution of eFluor450 as a measure of the proliferation of the transferred cells (right). **(f)** The proportion of Vα3.2^+^ Vβ11^+^ T cells in wild type (WT, n = 5) and CD4^fs^ (n = 6) mice. **P = 0.0043 (Mann Whitney’s U test). Each circle represents one mouse. Data represented as median with 95% CI. The experiments were performed twice with similar results.

To identify the unknown genetic mutation responsible for the CD4-deficient phenotype, we performed whole-exome sequencing. Comparison of the whole-exome sequencing data with the reference C57BL/6 genome sequence identified several indels and nonsynonymous coding variants throughout the genome. Interestingly, one coding variant was located within the exon 7 of the CD4 gene itself and this is a single nucleotide deletion at the codon 391 (p.Val391fs/c.1171delG; NM_013488.2) that resulted in a frameshift mutation creating a premature stop codon (Fig 2a). Hence, we refer to this new mouse strain as CD4^fs^ mice. The stop codon was upstream of the transmembrane domain of CD4, presumably resulting in a truncated CD4 protein that cannot be transported to the plasma membrane. We confirmed the presence of single nucleotide deletion at this region by Sanger sequencing of mRNA isolated from the thymus as well as tail genomic DNA (Fig 2b).

### The CD4 frameshift mutation abolished transmembrane CD4 expression

To understand the consequence of this frameshift mutation, we performed experiments to determine whether the mutant gene affects the localization of the CD4 co-receptor on the cell membrane. We cloned the frameshift mutant and wild-type allele of CD4 into a bi-cistronic retroviral expression vector which also expresses blue fluorescent protein (BFP) separated by an internal ribosome entry site (IRES) sequence. In this expression system, both CD4 and BFP genes are controlled by the same retroviral promoter, making BFP expression a proxy marker for the expression of the CD4 gene located upstream. We prepared the retrovirus and infected the T cell hybridoma 58 –/–cell line which does not express CD4. After retroviral transduction, we found more than 70% of the transduced cells express BFP (Fig 2c). However, when we compared the surface expression of CD4, we found that CD4 expression was observed in cells transduced with the wild-type CD4 construct but not in cells transduced with the CD4-mutant construct (Fig 2c). To prove that this frameshift mutation is responsible for defective CD4 expression, we set out to correct the mutation by CRISPR knock-in approach. We sorted BFP^+^ cells from cells transduced with the CD4 mutant construct and established a stable line with 100% expression of BFP. We then electroporated these cells with a guide RNA targeting CD4 along with Cas9 and a repair oligonucleotide template that contains wild type CD4 sequence. While the untreated cells did not show membrane CD4 expression, approximately half the cells that were transfected with Cas9/sgRNA ribonucleoprotein complexes expressed CD4 on the cell surface (Fig 2d). Thus, we concluded that the frameshift mutation is responsible for the defective CD4 T cell development in this mouse strain.

### CD4^fs^ mice are suitable for adoptive T cell transfer studies

Adoptive cell transfer studies are an important experimental paradigm to study CD4^+^ T cell functions *in vivo*. This is typically performed by transferring CD4^+^ T cells into either wild-type, congenic strains that can distinguish host and transferred cells or in RAG-deficient mice. While these paradigms have some advantages, they are not ideal for the exclusive study of the specific T cell population. For instance, the transfer of T cells into wild-type or congenic host would result in a mixture of host and transferred T cells in the lymphoid organs. Further, the host T cells would also respond to subsequent treatments such as immunization that may have an impact on the transferred cells thus making it difficult to distinguish intrinsic donor cell responses. Similarly, transfer of polyclonal T cells into RAG-deficient mice which lack CD4^+^ T cells CD8^+^ T cells, and B cells, would result in uncontrolled homeostatic proliferation. Thus, a mouse strain that exclusively lacks CD4^+^ T cells but no other population would be a better recipient for the adoptive transfer of CD4^+^ T cells. To demonstrate the suitability of this CD4^fs^ mouse strain for adoptive transfer studies, we transferred myelin antigen MOG-specific T cells into these mice and subsequently immunized them with MOG 35–55 peptide together with complete Freund’s adjuvant. Analysis of lymph node T cell composition showed that the CD4 T cell compartment in the CD4^fs^ mice contained exclusively the transferred T cell population whereas only a minor fraction of wild-type lymph node cells contained transferred T cells. (Fig 2e and 2f, Table 1). Further, we found that the adoptively transferred T cells were able to respond to the antigen and proliferated extensively (Fig 2e). Thus, this model can be used for functional studies on individual CD4 cell subsets without influence from other subsets.

**Table 1:** Quantification of T cell numbers and frequencies from wild type and CD4^fs^ mice after adoptive transfer and immunization.

Group	Total CD4^+^ cells (% of CD45^+^)	MOG-specific T cells (% of CD4^+^)	MOG specific T cells (% of CD45^+^)	Total CD4^+^ T cells (count per million cells)	MOG specific-T cells (count per million cells)
CD4^fs^	0.7 ± 0.6	91.6 ± 5.1	0.6 ± 0.6	6679.1 ± 5511.1	6318.3 ± 5537.3
WT	25.5 ± 2.9	1 ± 0.4	0.3 ± 0.1	254709 ± 28857.9	2546 ± 1030.8

Flow cytometry analysis of inguinal lymph nodes from wild type (WT) or CD4^fs^ mice after adoptive transfer of 3–4 million eFluor450 labeled cells from myelin-specific TCR transgenic mice. Mice were immunized with 100 μg MOG 35–55 in 5 mg/ml of CFA 24 h after cell transfer and lymph nodes collected 72 h after immunization. The number and frequencies of the total CD4 population and the MOG specific CD4 T cells is shown as mean ± SD.

## Conclusion

Spontaneous de novo mutations are an important source of new alleles in a given population resulting in variants that can influence the risk of multiple diseases. Here, we discovered a spontaneous frameshift variant of the CD4 gene that arose in our maintenance mouse colony. This variant resulted in a loss of CD4 expression on the plasma membrane due to a premature stop codon resulting in the loss of the CD4 transmembrane domain. This new model exclusively lacks CD4^+^ T cells but no other population, thus making this model an effective tool for assessing CD4^+^ T cell function *in vivo*.
